# Impact of intraoperative lung-protective interventions in patients undergoing lung cancer surgery

**DOI:** 10.1186/cc7762

**Published:** 2009-03-24

**Authors:** Marc Licker, John Diaper, Yann Villiger, Anastase Spiliopoulos, Virginie Licker, John Robert, Jean-Marie Tschopp

**Affiliations:** 1 Department of Anaesthesiology, Pharmacology and Intensive Care, Faculty of Medicine, University of Geneva, rue Micheli-du-Crest, CH-1211 Geneva, Switzerland; 2 Clinique des Grangettes and Faculty of Medicine, University of Geneva, CH-1224 Geneva, Switzerland; 3 Biomedical Proteomics Group, Department of Structural Biology and Bioinformatics, Faculty of Medicine, University of Geneva, CH-1211 Geneva, Switzerland; 4 Department of Thoracic Surgery and Faculty of Medicine, University Hospital, CH-1211 Geneva, Switzerland; 5 Department of Internal Medicine, Chest Medical Centre, CH-3960 Montana and Faculty of Medicine, University of Geneva, CH-1211 Geneva, Switzerland

## Abstract

**Introduction:**

In lung cancer surgery, large tidal volume and elevated inspiratory pressure are known risk factors of acute lung (ALI). Mechanical ventilation with low tidal volume has been shown to attenuate lung injuries in critically ill patients. In the current study, we assessed the impact of a protective lung ventilation (PLV) protocol in patients undergoing lung cancer resection.

**Methods:**

We performed a secondary analysis of an observational cohort. Demographic, surgical, clinical and outcome data were prospectively collected over a 10-year period. The PLV protocol consisted of small tidal volume, limiting maximal pressure ventilation and adding end-expiratory positive pressure along with recruitment maneuvers. Multivariate analysis with logistic regression was performed and data were compared before and after implementation of the PLV protocol: from 1998 to 2003 (historical group, n = 533) and from 2003 to 2008 (protocol group, n = 558).

**Results:**

Baseline patient characteristics were similar in the two cohorts, except for a higher cardiovascular risk profile in the intervention group. During one-lung ventilation, protocol-managed patients had lower tidal volume (5.3 ± 1.1 vs. 7.1 ± 1.2 ml/kg in historical controls, *P *= 0.013) and higher dynamic compliance (45 ± 8 vs. 32 ± 7 ml/cmH_2_O, *P *= 0.011). After implementing PLV, there was a decreased incidence of acute lung injury (from 3.7% to 0.9%, *P *< 0.01) and atelectasis (from 8.8 to 5.0, *P *= 0.018), fewer admissions to the intensive care unit (from 9.4% vs. 2.5%, *P *< 0.001) and shorter hospital stay (from 14.5 ± 3.3 vs. 11.8 ± 4.1, *P *< 0.01). When adjusted for baseline characteristics, implementation of the open-lung protocol was associated with a reduced risk of acute lung injury (adjusted odds ratio of 0.34 with 95% confidence interval of 0.23 to 0.75; *P *= 0.002).

**Conclusions:**

Implementing an intraoperative PLV protocol in patients undergoing lung cancer resection was associated with improved postoperative respiratory outcomes as evidence by significantly reduced incidences of acute lung injury and atelectasis along with reduced utilization of intensive care unit resources.

## Introduction

Compared with other surgical procedures, thoracotomy is associated with the highest 30-day mortality rates, ranging from less than 1% for minor resections to up to 12% for pneumonectomies [1-3]. Postoperative onset of acute hypoxemia – unrelated to cardiac failure, pulmonary embolism, atelectasis, sepsis or bronchoaspiration – has attracted much interest as it has become the leading cause of death in patients undergoing lung resection [4,5]. The guidelines set forth by the American–European Consensus Conference on the acute respiratory distress syndrome have been widely adopted to describe this form of acute lung injury (ALI), previously coined postpneumonectomy pulmonary edema, low-pressure or low-permeability pulmonary edema [6].

Contrasting with other adverse cardiopulmonary events, the incidence of post-thoracotomy ALI has not shown any noticeable decrease although various treatment modalities such as noninvasive ventilation and nitric oxide inhalation have reduced the case-fatality rate [7,8]. Interestingly, a large tidal volume (V_T_) and an elevated inspiratory pressure during one-lung ventilation have been identified as strong predictors of ALI in two retrospective observational studies [9,10]. The hypothesis of ventilator-induced lung injury during one-lung ventilation has been further supported by the association between tidal volume exceeding 7 to 8 ml/kg predicted body weight (PBW) and the release of systemic and pulmonary inflammatory mediators [11]. Presently, the clinical benefits of lung-protective strategies using lower V_T _combined with positive end-expiratory pressure (PEEP) have been clearly demonstrated in randomized controlled trials including only critically ill patients with ALI/acute respiratory distress syndrome [12].

Considering the potential injurious effects of large tidal volume in patients with healthy lungs undergoing short-term one-lung ventilation, we hypothesized that adopting a protective lung ventilation (PLV) protocol as part of a collaborative quality improvement initiative would lead to further reduction in the incidence of post-thoracotomy ALI. In our institutional surgical database, we examined whether protocol-driven changes in ventilatory strategy initiated in 2003 were associated with better clinical outcomes compared with historical controls.

## Materials and methods

### Study design and settings

The retrospective cohort study was approved by the Institutional Research Board and included all consecutive cases of lung cancer resection performed in two affiliated medical institutions: an academic center (Hôpitaux Universitaires de Genève) and a tertiary reference hospital (Centre Valaisan de Pneumologie in Sion). As the study concerned retrospective analysis of data obtained during usual clinical practice, local regulations do not require written informed consent. All patients were operated on by one of two board-certified thoracic surgeons and were managed by the same team of cardiothoracic anesthesiologists.

Since 1 March 2003 the PLV strategy has been routinely implemented as a best-practice model for intraoperative management (PLV cohort, from March 2003 to March 2008). This PLV group entailed the application of low V_T _(< 8 ml/kg PBW), pressure-controlled ventilation, limitation of the inspiratory plateau pressure to 35 cmH_2_O, the addition of external PEEP (4 and 10 cmH_2_O) and performance of vital-capacity maneuvers (raising the inspiratory pressure up to 35 cmH_2_O for 7 to 10 seconds) at 30-minute intervals.

In our database we abstracted a comparison group of nonprotocolized consecutive patients undergoing operation during the preceding 5 years (1998 to 2003), these patients being referred as the historical control cohort. In this group, conventional volume-targeted ventilation was aimed to achieve V_T _of 9 to 12 ml/kg PBW during two-lung ventilation and of 8 to 10 ml/kg PBW during one-lung ventilation while avoiding inspiratory pressure exceeding 35 cmH_2_O; no recruitment maneuver was performed and PEEP was applied at the discretion of the attending anesthesiologist.

In both groups, the same anesthetic workstations were used (Dräger Primus or Zeus, Lübeck, Germany) with the respiratory rates and the oxygen inspiratory fraction adjusted to keep the end-tidal carbon dioxide between 4 and 6 kPa (30 and 45 mmHg) and to keep the arterial pulsed oxygen saturation above 90%.

The main outcome of interest was the development of ALI, defined according to the American–European Consensus Conference criteria as follows: sudden onset of respiratory distress; infiltrates on the chest radiograph consistent with pulmonary edema; impaired oxygenation with an arterial oxygen pressure-to-inspired oxygen fraction ratio (PaO_2_/FIO_2_ ratio) less than 300 mmHg for ALI; and absence of cardiac insufficiency or fluid overload, based on pulmonary arterial catheterization, echocardiogram and/or clinical evaluation [6]. Additional criteria for post-thoracotomy ALI included the onset respiratory distress within the first 48 hours after surgery. Patients presenting with aspiration of gastric contents, pneumonia, bronchopleural fistula or pulmonary embolism who later developed noncardiogenic pulmonary edema were considered secondary ALI patients if they fulfilled the American–European Consensus Conference criteria.

Secondary outcome variables were inhospital mortality, intensive care unit (ICU) admissions, duration of hospital stay as well as respiratory, cardiovascular and surgical complications (see Additional data file 1).

In a previous study, we reported a 4.2% incidence of post-thoracotomy ALI [10]. A sample size of 1,000 operated patients provided the power (80%) to detect a 50% relative risk reduction in post-thoracotomy ALI. The sample size therefore resulted from an *a priori *decision to limit the analysis to two consecutive periods, before and after implementing the PLV strategy, including at least 500 patients per group.

### Patients and perioperative management

Besides clinical evaluation, electrocardiography and laboratory screening, routine preoperative work-up included pulmonary function tests (Sensor Medics, Yorba Linda, CA, USA) with the lung diffusion capacity to carbon monoxide, lung biopsy, CT scan and/or positron emission tomography of the chest and abdomen. Patients with borderline spirometric results (forced expiratory volume in 1 second lower than 60% to 80% of the predicted value), impaired exercise tolerance or cardiac risk factors underwent complementary investigations (peak oxygen consumption, differential lung perfusion/ventilation scan, echocardiography, thallium myocardial scintigraphy and/or coronary angiogram).

After anesthesia induction, a left-sided double-lumen tube was inserted and its correct position was confirmed by fiberoptic bronchoscopy. Lung resection with systematic lymph node dissection was performed through an anterolateral muscle-sparing thoracotomy. Thoracic epidural anesthesia was initiated intraoperatively and continued postoperatively until chest-drain removal.

Intraoperatively, intravenous crystalloids were infused at a rate of 2 to 4 ml/kg/hour and blood losses were compensated with colloids and with red blood cell concentrates if the hemoglobin levels decreased below 80 to 90 g/l. All patients were extubated in the operating theater and were admitted to an intermediate care unit for at least 12 hours before being transferred to the surgical ward. During the first 48 hours after surgery, aerosolized salbutamol and ipratropium were routinely prescribed and a fluid balance of maximum 500 ml/day was targeted, by limiting oral and intravenous fluid intakes. A restrictive transfusion policy was adopted throughout both study periods, with transfusion triggers ranging between 80 and 95 g/l. Antimicrobial prophylaxis with cefazoline was administered for 24 hours.

### Data collection

Demographic, clinical, surgical and anesthetic data as well as perioperative complications were abstracted from a prospective registry including all patients who underwent thoracic surgery. These data were collected by study nurses, entered into the surgical database in the same manner during both study periods and were cross-checked for accuracy. Before surgical incision and 30 minutes after the start of one-lung ventilation, the following ventilatory data were recorded: V_T _(ml/kg PBW), inspiratory plateau pressure, PEEP and FIO_2_. The effective dynamic compliance was obtained by dividing the ventilator-delivered V_T _by the peak airway pressure minus the PEEP. Intraoperatively and postoperatively, the use of vasopressor drugs was recorded as well as the urine output and the amount of fluid intake (colloids, crystalloids and blood products). On the first day after surgery, arterial oxygen pressure (PaO_2 _in kPa) was measured using a blood gas analyzer (ABL-5 10 analyzer; Radiometer, Copenhagen, Denmark) and the PaO_2_/FIO_2 _ratio was calculated as the PaO_2_/FIO_2 _ratio. Postoperative complications were defined according to standard criteria (see Additional data file 1).

### Statistical analysis

For comparisons between the two cohorts, the unpaired Student *t *test was used for normally distributed data and the Mann–Whitney *U *test for non-normally distributed data. The Kolmogorov–Smirnov test was applied to decide whether the cohorts were normally distributed. The prevalence of risk factors and the incidence of complications in the two groups were compared by Fisher exact test. Multivariate logistic regression analysis using backward selection was performed to assess whether demographic, clinical, laboratory and surgical factors, fluid and ventilatory management were associated with the occurrence of primary ALI. We choose an inclusive cutoff value for the empiric level of significance (*P *< 0.2) at which we retained variables. The final model was assessed for goodness of fit using the Hosmer–Lesmeshow test and for omitted covariates and model misspecification using the link test [13]. All analyses were performed using SPSS software (version 14.0 for Microsoft Windows; SPSS, Chicago, IL, USA) and statistical significance was specified as a two-tailed type I error (*P *value) set below the 0.05 level.

## Results

Over a 10-year period, 1,091 patients underwent pulmonary resection for malignancy. Complete data were available in 533 patients from 1 March 1997 to 28 February 2003 and in 558 patients from 1 March 2003 to 28 February 2008.

As detailed in Table 1, baseline characteristics of patients were similar between the two cohorts – except for a higher cardiovascular risk profile in the PLV cohort, as evidenced by a greater prevalence of hypertension and diabetes mellitus along with more frequent prescription of cardiovascular drugs.

**Table 1:** Preoperative characteristics of the two cohorts of thoracic surgical patients

	Historical control cohort (n = 533)	PLV cohort (n = 558)	*P *value
Age	62 (12)	63 (12)	0.956
>70 years (%)	29	30	0.568
Gender, female (%)	35.6	36.9*	0.709
Body mass index (kg/m^2^)	25.1 (4.6)	25.3 (5.1)	0.567
Smoking (%)			
Current	66.2	63.8	0.724
Ex-smoker (>6 months)	10.1	11.3	0.884
Alcohol (>60 g/day)	13.1	14.2	0.686
ASA classes 3 and 4 (%)	42.2	48.4*	0.047
Comorbidities (%)			
Hypertension	24.4	35.1*	< 0.01
Coronary artery disease	8.4	9.7*	0.546
Heart failure	5.8	8.7*	0.066
Hypercholesterolemia	16.7	22.9*	0.013
Peripheral artery disease	7.5	7.6	0.951
Diabetes mellitus	9.6	10.7	0.045
Arrythmia	2.1	2.7	0.633
Conduction blockade	8.4	7.3	0.576
Stroke	2.4	2.8	0.602
Prior PTCA/CABGS (%)	2.6	4.3	0.179
Preoperative medications (%)			
β-Blockers	5.6	10.4*	0.006
ACE inhibitors/angiotensin II antagonists	11.1	16.5*	0.012
Statins	8.1	9.7	0.534
Corticoids	3.9	4.1	0.999
Antiplatelets	4.1	7.4*	0.032
Calcium channel blockers	3.7	4.1	0.874
Lung function			
Forced vital capacity (l/min)	3.51 (1.07)	3.49 (0.96)	0.885
Forced vital capacity (% predicted value)	95 (21)	92 (22)	0.798
FEV1 (l/min)	2.5 (1.1)	2.4 (0.9)	0.825
FEV1 (% predicted value)	82 (18)	81 (19)	0.912
Total lung capacity (l/min)	6.2 (1.4)	6.3 (1.8)	0.892
Total lung capacity (% predicted value)	102 (18)	101 (17)	0.921
Carbon monoxide diffusion capacity (% predicted value)	54 (14)	53 (13)	0.896
Laboratory data			
Hematocrit (%)	41.0 (5.1)	40.8 (4.9)	0.885
Creatinine clearance (ml/min)	83 (23)	85 (28)	0.387

Data presented as mean (standard deviation) or percentage. ACE, angiotensin-converting enzyme; ASA, American Society of Anesthesiologists; CABGS, coronary artery bypass graft surgery; FEV1, forced expiratory volume in the first second; PLV, protective lung ventilation; PTCA, percutaneous coronary angioplasty. *P < 0.05 between the two groups.

The type of surgery, the distribution of pathological cancer stages, the need for chemoradiotherapy as well as the duration of one-lung ventilation and surgery did not differ between the groups (Table 2). Fluid and vasopressor therapies were also similar; however, a higher proportion of patients received continuous thoracic epidural anesthesia in the PLV cohort compared with the historical controls (98.2% vs. 92.3%, *P *< 0.05).

**Table 2:** Perioperative surgical and medical characteristics

	Historical control cohort (n = 533)	PLV cohort (n = 558)	*P *value
Preoperative chemotherapy	10.8	14.4	0.080
Type of surgery (% cases)			
Pneumonectomy or bi-lobectomy	21.4	17.6	0.129
Lobectomy	54.2	56.1	0.575
Lesser resection	18.7	20.5	0.542
Explorative thoracotomy	5.4	3.2	0.099
Pathologic stage (% patients)			
Ia and Ib	42	44	0.531
IIa and IIb	22	23	0.750
IIIa	18	19	0.733
IIIb and IV	11	8	0.389
Other	7	6	0.833
Thoracic epidural analgesia (% patients)	83.7	95.0*	< 0.001
Intraoperative period			
Duration of anesthesia (minutes)	166 (62)	176 (74)	0.228
Duration of surgery (minutes)	114 (47)	121 (56)	0.354
Duration of one-lung ventilation (minutes)	71 (18)	74 (20)	0.421
Total intraoperative fluid intake (ml/kg/hour)	5.6 (2.8)	5.8 (2.9)	0.587
Intravenous crystalloids (ml/kg/hour)	3.6 (1.4)	3.5 (1.6)	0.652
Intravenous colloids (ml/kg/hour)	2.0 (1.5)	2.3 (1.8)	0.187
Red blood cell transfusion (% patients)	1.50	1.61	0.991
Urine output (ml/kg/hour)	1.8 (1.2)	1.5 (1.4)	0.287
Phenylephrine (mg)	528 (443)	588 (487)	0.338
Ephedrine (mg)	15 (13)	16 (14)	0.542
Cumulative fluid intake (24 hours) (ml/kg/hour)	6.4 (3.2)	6.6 (3.1)	0.385
Postoperative period			
Total fluid intake (ml/24 hours)	1,657 (573)	1,496 (608)	0.182
Red blood cell transfusion (% patients)	2.25	1.79	0.890
Urine output (ml/24 hours)	868 (334)	781 (234)	0.254
Chest drainage (ml/24 hours)	378 (172)	342 (125)	0.774
PaO_2_/FIO_2 _(kPa)	45.1 (6.2)	44.7 (5.9)	0.513
Blood hemoglobin at POD1 (g/l)	119 (13)	121 (20)	0.834
Serum creatinine at POD1 (mg/l)	93 (30)	84 (31)	0.303

Data presented as mean (standard deviation), *n *(%) or percentage. PaO_2_/FIO_2_, ratio of arterial oxygen pressure to inspiratory fraction of oxygen; PLV, protective lung ventilation; POD1, first postoperative day. *P < 0.05 between the two groups.

During one-lung ventilation V_T _< 8 ml/kg was achieved in 92% of protocolized PLV patients (vs. 24% in historical controls), resulting in significantly lower V_T _and inspiratory plateau pressure, while the dynamic compliance, PEEP and respiratory rate were significantly higher compared with the historical control cohort (Table 3).

**Table 3:** Intraoperative Ventilatory management

	Historical control cohort (n = 533)	PLV cohort (n = 558)
Two-lung ventilation		
Tidal volume (ml/kg predicted body weight)	9.2 (2.8)	6.5 (2.0)*
Patients with tidal volume < 8 ml/kg (%)	24	92 *
Inspiratory plateau pressure (cmH_2_O)	16 (5)	12 (4)*
Positive end-expiratory pressure (cmH_2_O)	3 (2)	3 (3)
Dynamic compliance (ml/cmH_2_O)	52.4 (9.1)	53.5 (8.7)
Inspiratory oxygen fraction (%)	40 (4)	38 (13)
Respiratory rate (cycle/minute)	11 (1)	14 (2)*
One-lung ventilation		
Tidal volume (ml/kg predicted body weight)	7.1 (1.2)	5.3 (1.1)*
Inspiratory plateau pressure (cmH_2_O)	20 (7)	15 (6)*
Positive end-expiratory pressure (cmH_2_O)	3.3 (2.1)	6.2 (2.4)*
Dynamic compliance (ml/cmH_2_O)	32.2 (7.5)	44.6 (6.9)*
Inspiratory oxygen fraction (%)	64 (9)	67 (8)
Respiratory rate (cycle/minute)	13 (2)	15 (2)*

Data presented as mean (standard deviation) or percentage. *P < 0.05 between the two groups.

In the PLV cohort there was a reduction in the frequency of post-thoracotomy ALI (from 3.7% to 0.9% in the historical control cohort; *P *< 0.01) along with a lower incidence of atelectasis, fewer admissions to the ICU and a shorter hospital stay (Table 4). In the control cohort, patients ventilated with V_T _< 8 ml/kg presented a trend for a lower rate of ALI (0.8% vs. 4.9% in patients ventilated with V_T _>8 ml/kg, *P *= 0.08).

**Table 4:** Postoperative outcomes of patients undergoing lung cancer resection

	Historical control cohort (n = 533)	PLV cohort (n = 558)	*P *value
Length of hospital stay (days)	14.5 (3.3)	11.8 (4.1)*	< 0.001
Admission to the intensive care unit (%)	9.4	2.5*	< 0.001
Mortality (%)	2.8	2.3	0.753
Reoperation (%)	1.0	1.6	0.687
Respiratory complications (%)	14.4	10.8	0.080
Atelectasis	8.8	5.0*	0.018
Pneumonia	5.6	4.1	0.309
Bronchopleural fistula	1.5	1.3	0.873
Acute lung injury	3.8	0.9*	0.032
Pneumonectomy	10.7	3.1	0.094
Lobectomy, bi-lobectomy	1.7	0.2	0.174
Lesser resection	3.8	0.7	0.282
Mechanical ventilation >24 hours	4.1	3.5	0.379
Cardiovascular complications (%)	12.0	11.3	0.723
Myocardial infarct	1.3	0.9	0.711
Heart failure	0.9	1.4	0.635
Stroke	0.8	0.7	0.951
Arrhythmia's	11.8	10.4	0.514
Renal dysfunction	5.1	3.0	0.123

Data presented as mean (standard deviation) or percentage. *P < 0.05 between the two groups; χ^2 ^test with Yates correction or unpaired Student *t *test.

The cause of death was primarily attributed to ALI in one out of five patients in the PLV group (vs. 6/20 in the historical controls), other causes being related to sepsis (2/5 vs. 4/20, respectively), thromboembolism (1/5 vs. 3/20, respectively) and myocardial infarct (1/5 vs. 1, respectively). Inhospital mortality and the incidence of cardiovascular complications and secondary ALI did not differ between the two groups.

When adjusted for baseline characteristics and perioperative nonrespiratory management, the PLV intervention was associated with a decreased likelihood of ALI occurrence (adjusted odds ratio = 0.34 with 95% confidence interval = 0.23 to 0.75; *P *= 0.002). As detailed in Table 5, multivariate logistic regression analysis identified other independent risk factors for ALI: the extent of lung resection (pneumonectomy, adjusted odds ratio = 2.52 with 95% confidence interval = 1.34 to 7.71), V_T _(adjusted odds ratio = 1.17 per ml/kg increase with 95% confidence interval = 1.02 to 1.26), alcohol consumption (exceeding 60 g per day, adjusted odds ratio = 1.93 with 95% confidence interval = 1.14 to 5.71) and the cumulated amount of perioperative fluid infused (adjusted odds ratio = 1.42 per 1 ml/kg/hour increase with 95% confidence interval = 1.09 to 4.32). There was no evidence that additional covariates would improve the model (*P *= 0.21 by the Wald link specification test). The *c*-index for this model was 0.64 and the Hosmer–Lemeshow test for lack of fit was not significant (*P *= 0.56).

**Table 5:** Variables associated with post-thoracotomy acute lung injury

Characteristic	Unadjusted analysis		Adjusted analysis	
		
	Odds ratio (95% confidence interval)	*P *value	Odds ratio (95% confidence interval)	*P *value
Age, per 10-year increase	1.09 (0.80 to 1.89)	0.382	-	-
Chronic alcohol consumption	1.76 (1.11 to 5.2)	0.013	1.93 (1.14 to 5.71)	0.001
FEV1 < 60%	1.12 (0.78 to 2.05)	0.254	-	-
ASA class 3/4	1.21 (0.72 to 2.21)	0.214	-	-
ACE inhibitor therapy	0.85 (0.55 to 2.12)	0.315	-	-
Statin therapy	0.81 (0.45 to 2.97)	0.198	-	-
Chemo-radiotherapy	1.52 (1.09 to 3.83)	0.021	1.40 (0.91 to 2.98)	0.203
Advanced TNM stages (III to IV)	1.63 (1.09 to 3.01)	0.018	1.45 (0.87 to 2.84)	0.234
Thoracic epidural anesthesia	0.92 (0.78 to 1.92)	0.563	-	-
Duration of surgery	1.37 (0.78 to 2.67)	0.312	-	-
Red blood cell transfusion	1.09 (0.23 to 7.24)	0.789	-	-
Pneumonectomy	2.41 (1.29 to 8.12)	0.005	2.52 (1.34 to 7.71)	< 0.001
Fluid infused, per 1 ml/kg/hour increase	1.33 (1.02 to 5.08)	0.032	1.42 (1.09 to 4.32)	0.011

ACE, angiotensin-converting enzyme; ASA, American Society of Anesthesiologists; FEV_1_, forced expiratory volume in 1 second; TNM, Tumor, Node, Metastasis.

## Discussion

The present observational study is the first to indicate that implementation of an intraoperative ventilatory strategy aimed to limit lung overdistension while maintaining functional residual capacity with external PEEP and recruitment maneuvers leads to significant reduction in the incidence of post-thoracotomy ALI and atelectasis along with fewer admissions to the ICU and a shorter hospital stay.

Importantly, lowering the risk of ALI with PLV by more than 50% was independent of age, severity of underlying lung and cardiovascular diseases as well as other perioperative interventions. Although the present results were obtained by comparison with a historical control group, they strongly suggest that the PLV strategy may also benefit patients undergoing lung cancer resection. Alternatively, the improved respiratory outcome in PLV-treated patients supports the hypothesis that ALI and atelectasis may in part be caused by or be related to intraoperative factors: ventilator-induced lung injury or ventilator-associated injuries and the reduction of functional residual capacity consequent to the effects of surgical insults, anesthesia and muscle paralysis [14,15]. High shear stress associated with cyclic opening of collapsed areas (atelectotrauma) and deformation of the alveolar epithelium (strain) during one-lung ventilation are thought to generate a proinflammatory state (biotrauma) leading to pulmonary tissue alterations.

By the late 1990s the standard V_T _for managing thoracic surgical patients had already been adjusted downwards (from 10 to 12 ml/kg in the 1980s) to 8 to 10 ml/kg, although no specific guidelines existed for one-lung ventilation. Our historical control data were consistent with these values and, after implementation of the PLV protocol, the V_T _declined from mean values of 7.1 to 5.3 ml/kg during the one-lung ventilation period. We used predicted rather than actual body weight for calculating the V_T_ per kilogram of body weight to avoid lung overdistension in obese patients and in women who have smaller lung volumes [16]. Importantly, the ventilatory endpoints (V_T _< 8 ml/kg and inspiratory plateau pressure < 35 cmH_2_O) were achieved in more than 80% of patients. Compliance with the new ventilatory guidelines was facilitated by the relatively short ventilatory time (< 3 hours), the absence of acute critical illnesses and the commitment of a small number of cardiothoracic anesthesiologists. Interestingly, similar protective ventilatory strategies applied in ICU settings have been associated with a decreased incidence of ALI in high-risk patients, although the target V_T _was achieved in only 50% to 60% of cases [17-19].

Thoracic surgical candidates represent a particular group of noncritically ill patients in whom ventilation-induced cytokine upregulation produces a proinflammatory state that renders the host more vulnerable to subsequent hit(s) such as ischemia–reperfusion, hypoxia–reoxygenation and direct tissue trauma [20-22]. Depletion of pulmonary glutathione stores observed in alcoholic patients is expected to further exacerbate oxidative lung injuries [23].

To date, three randomized controlled trials including patients undergoing thoracotomy have compared the application of traditional high V_T _with the open lung strategy combining low V_T _and PEEP. Although Wrigge and colleagues failed to document any difference in systemic inflammatory markers [24], Schilling and colleagues found reduced alveolar concentrations of TNFα and soluble intercellular adhesion molecules in patients ventilated with small V_T _(5 vs. 10 ml/kg) [25]. Confirming these positive results, Michelet and colleagues reported an attenuated systemic proinflammatory response, lower interstitial pulmonary edema and an improved oxygenation index allowing earlier extubation in the protective ventilation group among patients undergoing esophagectomy [26].

In the present study we adopted a PLV including pressure-controlled ventilation, external PEEP and recruitment maneuvers. Actually, delivery of a decelerating gas flow has been reported to achieve more homogeneous flow distribution and lower peak airway pressure [27]. Different lung recruitment strategies have been shown to re-expand the collapsed dependent lung areas that develop in almost all anesthetized patients. During thoracic surgery, application of recruitment maneuvers with moderate PEEP levels to the dependent lung has been shown to improve oxygenation and to reduce both intrinsic PEEP levels and static elastance of the respiratory system without causing significant cardiovascular deterioration [28]. Our data confirm the good hemodynamic tolerance to the PLV protocol since fluid and vasopressor requirements were similar in the two cohorts. Given the difficulties in constructing static pressure–volume curves, we did not titrate the PEEP but we set a fixed moderate level of PEEP that could potentially cause alveolar hyperinflation in healthy or emphysematous areas. This possibility seems unlikely since we observed higher compliance in patients managed with the PLV protocol, which supports the stabilizing effects of PEEP along with effective re-expansion of previously collapsed areas following recruitment maneuvers [29-31].

We acknowledge several limitations in the current study. Although data were collected by clinicians and validated by scientific investigators, we assume variability in initial ventilator settings with the possibility that higher inspiratory pressures and tidal volume were deliberately chosen to correct transient hypoxemia and hypercapnia. The observational design limits the ability to infer causality between the lung-protective protocol and lowering the incidence of ALI. Although statistics were helpful to adjust for some confounding variables, unmeasured factors and other changes in practice or in the patient case mix may have decreased the confidence in observed effects. For instance, potentially beneficial therapies such as preoperative statin and angiotensin-converting enzyme treatment, continuous thoracic epidural anesthesia, goal-directed fluid therapy using transesophageal Doppler monitoring, inhalation of β_2_-agonists in high-risk patients, and early postoperative mobilization were popularized during the postintervention period, and thereby could have contributed to the overall reduction in respiratory complications and in hospital length of stay [32]. On the other hand, despite higher prevalence of hypertension in the protocol-treated cohort, mortality and cardiovascular adverse events were unchanged compared with the control cohort. Finally, major limitations also stem from the definition of ALI that may cover different clinical patterns and histological findings, which may explain significant interobserver diagnostic disagreement particularly in postoperative patients [33]. In the present study, we excluded patients with delayed onset of ALI triggered by infection, bronchial aspiration of gastric content and allogenic transfusion. Accordingly, post-thoracotomy ALI probably identified a more homogeneous group of patients predisposed to the injurious effects of mechanical ventilation. The reliability of ALI diagnostic criteria could have been improved by additional measurements of plasma brain natriuretic factor and lung water content with the transpulmonary thermodilution technique [34,35].

## Conclusions

In this observational study, we demonstrated the effectiveness of combining low V_T_, PEEP and recruitment maneuvers. This intraoperative open-lung approach was easily implemented in clinical practice and resulted in a reduced incidence of postoperative ALI and atelectasis. Implementation of a bundle of scientifically-based perioperative interventions represents an integral component of clinical quality management. Future clinical trials will determine whether optimization of other ventilator settings (for example, oxygen inspiratory fraction, PEEP level, periodicity of recruitment maneuver) may improve respiratory outcome in specific groups of surgical patients requiring mechanical ventilation.

## Key messages

• Intraoperative application of a small tidal volume, PEEP and recruitment maneuvers was successfully achieved in 92% patients undergoing lung cancer resection over a 5-year period.

• Adoption of the presented lung ventilatory strategy was associated with a reduced incidence of acute lung injury (0.9% vs. 3.7%) and atelectasis (5% vs. 8.8%), and with fewer admissions to the ICU (2.5% vs. 9.4%) and a shorter length of hospital stay (11.8 ± 4.1 vs. 14.5 ± 3.3 days).

• Traditional intraoperative ventilatory settings can be harmful, and therefore new guidelines should be proposed.

## Abbreviations

ALI: acute lung injury; ICU: intensive care unit; PaO_2_/FIO_2 _ratio: oxygenation index, ratio of arterial oxygen pressure to inspired oxygen fraction; PBW: predicted body weight; PEEP: positive end-expiratory pressure; PLV: protective lung ventilation; TNF: tumor necrosis factor; V_T_: tidal volume.

## Competing interests

The authors declare that they have no competing interests.

## Authors' contributions

ML and J-MT participated in the study design, data analysis and interpretation of the data as well as the writing of the manuscript. JD, VL and ML participated in the data collection and statistical analysis. JD, YV and JR participated in the literature search and interpretation of the study. AS and JR participated in revising the bibliography, and correcting and editing the manuscript. All authors read and approved the final manuscript.

## Supplementary Material

Additional file 1 A word file containing a table that lists the major nonfatal complications occurring during the inhospital postoperative stay. Standard criteria are used to define these adverse events. Click here for file
