# Supplementary material for: Impact of intraoperative lung-protective interventions in patients undergoing lung cancer surgery
Source: Crit Care. 2009 Mar 24;13(2):R41. doi: 10.1186/cc7762 (PMC2689485; doi:10.1186/cc7762)
Supplement: Additional file 1 — A word file containing a table that lists the major nonfatal complications occurring during the inhospital postoperative stay. Standard criteria are used to define these adverse events. [file cc7762-S1.doc]

**Additional data file 1**

This table lists the major non-fatal complications occurring during the in-hospital postoperative stay. Standard criteria are used to define these adverse events.

**Major non-fatal complications**

### Cardiovascular

1. Myocardial infarct : typical rise and fall of CPK (> 120 U/L) and CK-MB/CPK > 6% or troponin-I > 1.5 ng/ml with at least one of the following criteria: ischemic symptoms, development of pathological Q waves on the ECG, ST segment elevation or depression (> 1 mm) or coronary artery intervention
2. Arrhythmia’s : supraventricular and ventricular tachyarrhythmia's on ECG requiring anti-arrhythmic medications and/ or an electrical cardioversion
3. Congestive heart failure : need for sympathomimetic support, diuretics or vasodilators consistent with clinical, hemodynamic (pulmonary artery pressure > 15 mmHg) and radiological evidence of pulmonary congestion

**Cerebral**

Stroke : focal neurological deficit (transient or permanent)

**Respiratory**

1. Atelectasis :Lobar collapse (Chest-X rays), need for CPAP and / or bronchoscopy
2. Bronchopneumonia: temperature > 38°C, hyperleucocytosis (neutrophils), new lung infiltration (chest-X rays), positive culture (bronchial secretions or alveolar fluid)
3. Prolonged mechanical ventilation > 24h

**Renal**

Renal dysfunction: elevation of serum creatinine > 20% compared with preoperative values
